# Decision-making under uncertainty in environmental health policy: new approaches

**DOI:** 10.1186/s12199-019-0813-9

**Published:** 2019-09-14

**Authors:** Jacques Reis, Peter S. Spencer

**Affiliations:** 1 Faculté de Médecine, University of Strasbourg, 4 Rue Kirschleger, 67000 Strasbourg, France; 2 Association RISE, 3 rue du loir, 67205 Oberhausbergen, France; 3 Oregon Institute of Occupational Health Sciences and School of Medicine (Neurology), Oregon Health & Science University, Portland, OR 97201 USA

**Keywords:** Uncertainty, Decision-making, Environmental health policy, Scientific doubt, Stakeholders, Policy-makers, Ethical issues, Scientific communication

## Abstract

Decision-making in environmental health policy is a complex procedure even in well-known conditions. Thus, in the case of uncertainty, decision-making becomes a hurdle race. We address scientific uncertainty, methods to reduce uncertainty, biomedical doubt and science communication, and the role of stakeholders, activists, lobbies and media that together influence policy decisions. We also consider the major responsibility and role of the medico-scientific community in this process. This community can and should teach the principle of scientific uncertainty to all stakeholders, advise policy-makers and underline the ethical issues, considering that our brains are not only the deposit of our humanity but also the route to environmental health and societal harmony.

## Introduction

Societies generally agree that environmental pollution is a key concern for human health. This has led citizens in many parts of the world to question relevant policies proposed by top decision-makers. Numerous scientists and physicians are likewise surprised by the actions and inactions of some policy-makers at national and international levels. A chasm sometimes seems to exist between knowledge presented by the medico-scientific community and the responses of policy-makers. The biomedical community has a special responsibility to address this problem because it generates data that define the nature and advance of environmental pollution and, by extension, the quality of human health from conception to death.

In the context of global environmental health, we address scientific uncertainty, methods to reduce uncertainty, biomedical doubt and science communication, and the role of lobbies, stakeholders and media. We discuss decision-making procedures used in other fields of knowledge and offer suggestions intended to help solve the dilemma of insufficient knowledge and scientific uncertainty that can influence policy decision-makers.

## Scientific uncertainty

As life in general, scientific and medical knowledge is paved with uncertainty. Thus, the French philosopher and sociologist Edgar Morin advises in his chapter on confronting uncertainties: “We should learn to navigate on a sea of uncertainties, sailing in and around islands of certainty” [1]. As uncertainty is the major challenge in the fields of anticipation, prospection and prevision, researchers have classified uncertainty into four levels. In type 1, deterministic laws apply, allowing facile anticipation: there is no uncertainty. In the case of type 2 uncertainty, stochastic methods (e.g. Markov’s chains) can assess the probability of an event. Type 3 combines a qualitative certainty and quantitative uncertainty; for example, personal age of death is an event ruled by probabilistic laws. Type 4 is the highest uncertainty level as it combines both qualitative and quantitative characteristics, e.g. earthquake, tsunami and volcanic eruption, among other examples [2].

Typically, matters relating to environmental health fall into a type 4 case. Here, uncertainty is related to complexity, which results from a multitude of interacting chemical, physical and/or societal forces, coupled with their interactions with multiple exogenous and endogenous factors, the latter exemplified by stress. Furthermore, for the medico-scientific community, uncertainty is hallmarked by caution, rigorous adherence to available data, and a conservative interpretation thereof. Thus, a probabilistic approach to causation is often the only rational and honest conclusion. Ignorance, lack of knowledge, or cognitive limitations of complex phenomena (e.g. natural hazard, industrial risk, infectious disease, social disruption, population susceptibility) are common and hinder an unequivocal interpretation of available data. Given these considerations, uncertainty, meaning “scientific doubt”, is a very useful doctrine for biomedical scientists engaged in environmental health and many other disciplines. Therefore, on the one hand, uncertainty is a positive concept that stimulates the creation of testable hypotheses and drives research, for example on the role of natural and synthetic chemicals in the genesis of neurodegenerative diseases [3]; on the other, uncertainty must be evaluated and mitigated. Nevertheless, when using precise axioms and methods, medico-scientific knowledge is a sound basis for decision-making in environmental health.

## Scientific methods and expertise

Scientific knowledge and expertise are the core of the decision-making process for all governmental regulatory bodies that address environmental health, including causality in complex interactions, risk assessment and risk management [4–6]. Reducing the level of uncertainty in causality and risk assessment is imperative. Many helpful methods have been developed over the past 50 years, notably in medicine, epidemiology and toxicological risk assessment. These include the Bradford Hill criteria [7], the methods developed by the International Agency for Research on Cancer (IARC) [8], the clinical “gold standard” of evidence-based medicine that is also used in public health [9] and the experimental verification of chemical cause and health effect.

Epidemiological and cohort studies constructed on a solid foundation of deep investigative enquiry that generates testable hypotheses provide a critical springboard for further enquiry. In 1954, Sir Austin Bradford Hill famously proposed his nine criteria for assessing chemical causation to help interpret findings that connected cigarette smoking with lung cancer [10] even though he emphasized that cause–effect decisions cannot be simply rule-based [11]. The IARC has underlined the importance of the strength of available evidence (notably coming from meta-analyses and pooled analyses) and the robustness of cohort and case–control studies in assessing cancer risks from chemical exposures [8]. Several countries have created large cohorts for long-term prospective study, data from which have revealed robust evidence of the specific health risks, notably for children, such as now well-accepted link between prenatal and early-life harmful environmental exposure and health impact, in both childhood and adulthood [12–15].

Experimental verification of cause–effect hypotheses based on associations discovered in epidemiological studies is indispensable. Statistically significant results of appropriately designed and rigorously controlled research, whether conducted in silico, at the molecular, cellular or whole-animal level, are critical components for environmental risk assessment. Quantitative structure–activity relationships (QSARs) to predict ecologic effects and the environmental fate of chemicals [16] as well as QSARs and structure–activity relationships (SARs) to predict toxicity, mutagenicity, carcinogenicity and other health effects [17], are used by regulatory agencies to assess health risks. More recently, these approaches have been supplemented by big-data processing, machine learning, artificial intelligence [18, 19], and enhanced by the concept of the exposome [20], a representation of the complex lifetime exposures to chemical, physical and other exogenous factors.

Once armed with the weight of evidence from experimental verification of hypotheses developed from population-based studies, we face critical decisions before proceeding with the implementation of a scientific-based response. The first is the level of proof and the acceptable degree of persistent uncertainty (near-certainty?) that can be tolerated. The second relates to “proof hierarchy”, given that perception of the accuracy of the level of proof differs among diverse fields of biomedical knowledge. There is still a debate about the relative strength of different types of evidence (e.g. from clinical cases, epidemiological associations, experimental data) generated by scientists and clinicians in search of disease aetiology. To surmount these issues in assessing data, an organization such as IARC convenes international, interdisciplinary working groups of expert scientists to review systematically the pertinent scientific literature and to develop consensus evaluations and classifications. IARC selects these experts based on their knowledge and experience and the absence of real or apparent conflicting interests. A similar approach is employed by the Board on Environmental Studies and Toxicology of the National Academies (http://dels.nas.edu/best), an august non-governmental body in the USA.

The approach taken by such organizations can be viewed as stellar and unimpeachable since the resulting reports on hazardous environmental factors represent health-related projections based on careful, expert and unbiased assessment of available data. While such approaches seek to identify and quantify environmental exposures that constitute long-term health risks, they are unable for obvious reasons to state future health effects with certainty. This is a major limitation and concern as it relates, for example, to foetal development, brain function [21], reproductive health and other critically important properties of the human condition. The question must therefore be posed as to whether there are other approaches with the potential to mitigate critical health risks.

## Decision-making in strategic activities with critical risks

Decision-making is such a strategic component of many common human activities that this has led to the development of decision theory, “which gives tools for making rational choices in the face of uncertainty” [22–24]. This approach is used not only in medicine [25, 26] but also in technology, politics, defence, economics and the management of infrastructure and major natural risks. Can environmental health policy experts benefit and learn from the concepts that have emerged in non-health professions?

The US National Aeronautics and Space Administration (NASA) developed a new concept to address risks, namely Criticality Analysis and Risk Assessment (CARA). This bottom-up inductive analytical process is based on possibilities for system failures and the severity of the effects thereof. Thus, the criticality is the combined measure of the relationship between the probability of failure (PoF) and the consequence of failure (CoF) of a particular asset. The key parameters are vulnerability of the targets, the possible range of system failures, their probability of occurrence, and the consequences thereof. Sequential analytical steps include asset inventory, the PoF and CoF of each asset, and a weighting reflecting asset value, all of which are individually combined into an asset-specific criticality index. Critical and non-critical assets are identified, and interventions are designed and adopted to protect the former [27, 28]. As applied to the human condition, while the heart, lungs, liver and other organs are critical assets, we suggest the human genome and brain are at the zenith.

Another example of risk analysis is the Catastrophe Theory of French (René Frédéric Thom) and Russian (Vladimir Igorevich Arnold) mathematicians. This is a mathematical exercise that uses theorems to predict sudden and discontinuous changes in diverse phenomena, such as the change from an intact to a collapsed bridge. In the domain of infrastructure and industrial plants, the theory is now used as the foundation for risk assessment and hierarchical risk management. It provides a method for the analysis of risk levels based on a multi-objective evaluation: inherent risk of the source, effectiveness of the prevention and control mechanism, and vulnerability [29].

The Joint Research Centre of the European Commission (EU) has provided an extensive review of the different effective risk assessment methodologies used in the European Union (EU) and worldwide (https://ec.europa.eu/home-affairs/what-we-do/policies/crisis-and-terrorism/critical-infrastructure_en). This is the foundation of the EU programme for Critical Infrastructure Protection (EPCIP), which seeks to provide an all-hazards cross-sectoral approach [30]. Identifying threats, assessing vulnerabilities and evaluating the impact on assets, infrastructure or systems, considering the probability of the occurrence of these threats, is indispensable. Japan’s 2011 Fukushima Daiichi disaster exemplifies the absolute need for an interprofessional multidisciplinary approach to risk assessment, viz. stratigraphic, paleontological and engineering expertise identified the Sendai area as prone to large 800–1000-year earthquake-triggered tsunami that deeply invade Pacific coastal areas of Central Japan [31].

Another example originates from the financial world. Business practice after the 2009 financial crisis employed a Volatility, Uncertainty, Complexity, and Ambiguity (VUCA) analysis to guide decision-making [32, 33]. Volatility of unknown duration and extent is addressed by risk-matched investment in human capital and preparedness, resources and inventory. Uncertainty is reduced by investment in information collection, interpretation and data sharing. Complexity is surmounted by recruiting information specialists and developing needed resources. Ambiguity acknowledges associations with no clear cause–effect relationships, which are addressed by generating and testing cognate hypotheses. In sum, VUCA is a conservative approach to perceived threats that is directly applicable to environmental health risk assessment. Stated otherwise, the quality of hazard assessment, whether it relates to the survival, resilience and long-term health of a business or of *Homo sapiens*, is directly related to the quality and amount of fundamental research, data collection, integration and interpretation.

Thus, in many fields, without any dissent or opposition, experts utilize diverse approaches that address and surmount problems posed by uncertainty. Perhaps one or more of these methods should be considered in Environmental Health. Should we advocate favouring a process that employs forward-looking, risk-based criticality analysis with the goal of preventing disease? Should regulations become proactive, albeit under an existing state of uncertainty? Is it reasonable to think in terms of the precautionary principle, which involves taking “preventive” action in the face of uncertainty, shifting the burden of proof to the proponents of an activity, exploring a wide range of alternatives to possibly harmful actions, and increasing public participation in decision-making [34–36]?

Do we have the appropriate international bodies to assess critical risks and develop a consensus on how to mitigate those risks?

The VUCA analysis dictates a conservative approach equivalent to the 1998 Wingspread Statement [37] on the precautionary principle: “When an activity raises threats of harm to human health or the environment, *precautionary* measures should be taken even if some cause and effect relationships are not fully established scientifically”. The 1987 Montreal Protocol and its sequential revisions relating to the release of ozone-depleting chemicals provide a concrete example of concerted international action taken under conditions of data uncertainty in favour of the precautionary principle. While it was only suspected by scientists that human use and release of chlorofluorocarbons and hydrofluorocarbons were slowly destroying the ozone layer, under United Nations leadership, two ozone-related treaties were ratified by 196 states and the European Union. Since then, approximately 98% of the ozone-depleting substances contained in nearly 100 hazardous chemicals have been phased-out worldwide, and the ozone layer appears to be in the process of recovery [38]. One can only hope that decisions initiated at the 2015 United Nations Conference on Climate Change will have a comparable positive outcome for human health and safety.

## Communication in science, risks and uncertainty, and stakeholders

When addressing public health issues, the common attitude of the medico-scientific community is to consider that the drive for discovery and application of new knowledge will provide answers to advance human health and wellness. The common way to draw public attention to advances is through scientific publication, the impact of which on decision-makers and the community-at-large is often limited. For example, when Philip Landrigan and Philippe Grandjean launched in *Lancet Neurology* their proposal for an international agency for research dedicated to the brain [39], no decision-making body expressed interest. In similar vein, despite media attention to the air pollution-focused 2018 World Brain Day organized by the World Federation of Neurology [40], the same year WHO ignored the brain and foetus during its first meeting on the health impact of air pollution. The social and environmental impact of scientific communication is thus a major unresolved issue [41–43]. Furthermore, scientific communication of new knowledge attended by uncertainty, to citizen, media and decision-makers, is challenging [44, 45]. Consider for example the discovery of global climate change, its geographically specific effects on maximum seasonal temperatures and their implications for human health. Application of this knowledge to public policy is obvious despite appropriate acknowledgement of inherent data uncertainty. Non-scientists, however, may be confused by this qualification, thereby allowing some leaders and policymakers to exploit the data uncertainty and use this bedrock scientific principle to question the accuracy and thereby diminish the significance of this immensely important observation, even though evidence suggests global warming may have existential significance for multiple species, including human groups. To bolster their position, naysayers can point to the dictionary definition of uncertainty, namely “an event or association for which there is doubt, scepticism, suspicion and even mistrust” [46]. The June 2017 announcement that the USA would withdraw from the Paris Climate Agreement provides a spectacular example.

It is easy for leaders, stakeholder lobbyists and supportive media to exploit scientific caution and use this to denigrate the judicious, evidence-based views of experts, albeit views that may be responsibly qualified by statements of uncertainty [47–50]. Support for their position is readily found in the form of psycho-facts (information not based on hard evidence but believed because of constant repetition) that are espoused by evangelical social engineers, including certain media, politicians, policy advocates and promoters of various causes and lifestyles [51]. Social movement activists may use fear to support their goals, which are not always scientifically backed and often based on emotion, not reason [52]. Such commonly employed tools can foster societal confusion, risk denial, and a lack of remedial or preventative action.

The biomedical community can and should act to reverse this troubling trend and false social perception in regard to matters of global health: we are required on ethical grounds to address key questions and bring to the debate clear and understandable information. Moreover, given society’s growing interest in ecological and personal health, sustained citizen education is needed to reduce knowledge gaps, counter “fake news” and expose data manipulation. We should explain the optimal methods by which environmental risks are assessed for human health significance. Mankind is challenged by many global threats (not only by global warming but also by hazardous chemicals in nature and of anthropogenic origin in soil, water, food and air) which with “mathematical” certainty have the capacity to modify in an irreversible manner our planet and life on Earth.

**Fig. 1:**
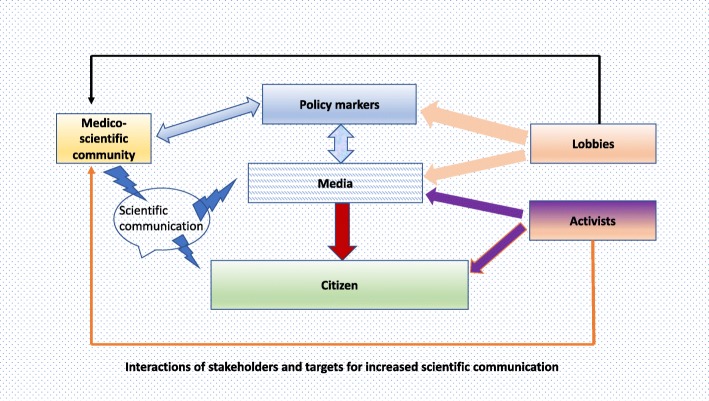
Interactions of stakeholders and targets for increased scientific communication

One of our key missions therefore is citizen education which, for successful impact, should consider possible interactions among key stakeholders (Fig. 1). The role of legal regulation, which can impede access to data [53], as well as the influence of media, advertisement bias and lobbyist pressure, have been scrutinized in several cases, including topics of finance [54] and health policy [55–57]. In environmental health policy, such studies need to address specific issues, such as pesticide use [58], albeit in a multidisciplinary manner. As with public nutrition, diet and/or hygiene recommendations associated with dissuasive taxation on consumer products (e.g. junk food with much salt and sugar, tobacco) can change personal behaviour and corporate strategy [59].

To reiterate, when considering policy and choices made by decision-makers, medico-scientific conclusions are often challenged by interested parties because of their underlying uncertainty. Furthermore, community policy often results from a multifaceted process, which considers several crucial issues at the same time, notably, the limits of science communication, lessons that flow from other human activities, and political and economic considerations, among others. Unfortunately, decision-makers often lack a scientific background, besides some rare personalities such as Margaret Thatcher, Angela Merkel and Xi Jinping, who have pushed for the adoption of new science-based ideas for innovative, coordinated, green, open and shared development. Given the widespread absence of scientific training among decision-makers, initiatives such as the former Harvard’s Center for Health and the Global Environment, are needed to increase awareness and understanding in ways that can promote global public health. Scientific and public pressure can be very effective: for example, 50+ years ago, a group of leading French public figures succeeded in persuading President Charles De Gaulle to address the ever-growing burden of cancer, a project that rapidly gained momentum and led to the creation of IARC in 1965. Former US Vice President Al Gore, founder of The Climate Reality Project, is an example of effective individual leadership that led to game-changing international action to reign in human activity driving global warming.

## Decision-making in environmental health: scientific arguments and ethical concerns

Public health decisions that result in policy must be taken, often at a precise moment, without the desirable full body of information that justifies a cause–effect conclusion and often for complex conditions. New ways to approach and solve these challenging decisions can include concepts such as criticality and risk-based criticality analysis. But is this enough?

History offers many examples of remarkable decisions taken under uncertainty (and, by today’s standards, minimal data) by famous members of the medico-scientific community. These include the following: Ignaz Semmelweis, who controversially used a hand antiseptic to control the spread of puerperal fever in Vienna in 1847 [60]; John Snow and the ban on use of the Broad Street water pump to control the London cholera outbreak of 1854 [61]; France’s Louis Pasteur and the first rabies vaccination in 1885 [62]; and Sir Alexander Fleming and the first antibiotic treatment with penicillium injections in 1929 [63].

Policy decisions that affect human health are taken mostly by governmental but also non-governmental organizations. Decisions can be disastrous because of a lack of multidisciplinary analysis: witness the well-meaning decision spearheaded by the United Nations and World Bank to create clean-water tube wells across Bangladesh, an initiative described by the World Health Organization as “the largest mass poisoning of a population in history” because the tubes well water serving an estimated 35–77 million people contain natural sources of arsenic in levels sufficient to produce arsenicosis linked to skin and other cancers, adverse pregnancy outcomes, sensory neuropathy and decreased intelligence quotient among children [64–67]. In other situations, it is the *lack* of decision that has provoked disasters. Examples include the 1952 Great Fog of London that resulted in 4000 fatalities in a few days, a result of failure to control air pollution first emphasized by John Evelyn in his 1661 *Fumifugium* pamphlet [68] and the disastrous 1930 Meuse Valley industrial fog in Belgium [69]. The opposite governmental policy approach is illustrated by one employed in Japan: In response to widespread deforestation and its ecological consequences, the Tokugawa shogunate during the Edo era (1603–1868) introduced a courageous policy based on restrictive and incentive regulations that led to forestry science and the sustainable use of wood. Thanks to this successful top–down policy, Japan was able to protect its forests [70].

The National Academies Institute of Medicine in Washington DC [26] and the European Environment Agency (EEA) [21] have reported historical case studies concerning environmental health policies, such as those regarding radiation and asbestos. The EEA has documented the lack of decision or the long delays before action was taken in response to early warnings and even to “loud and late” warnings. The historical analysis of more than thirty cases led to “late lessons”! The “Late Lessons Project” illustrates how damaging and costly the misuse or neglect of the precautionary principle can be, using case studies and a synthesis of the lessons to be learned and applied to maximizing innovations while minimizing harms [71].

Given that present-day societies face challenges that greatly exceed those in past centuries, new approaches are mandated for the development of policies that impact human health. First, risk assessment practice must engage broad multidisciplinary representation. Second, we must accept a level of uncertainty in all biomedical data but err on the side of the Precautionary Principle when it comes to the protection of global public health, especially for the foetus and the human brain, the seat of humanhood. This is not only responsible scientific practice in the service of Humanity, it is bolstered for physicians by the Hippocratic oath, an ethical commitment to the advancement of human health. As *Homo sapiens* is challenged by many global threats, which, with certainty, have the capacity to modify in an irreversible manner the future of humankind and other species of life on Earth, we urge policy makers to adopt the same ethic: *Primun non nocere—Above all, do no harm*.

## Data Availability

Data sharing is not applicable to this article as no datasets were generated or analysed during the current study.
